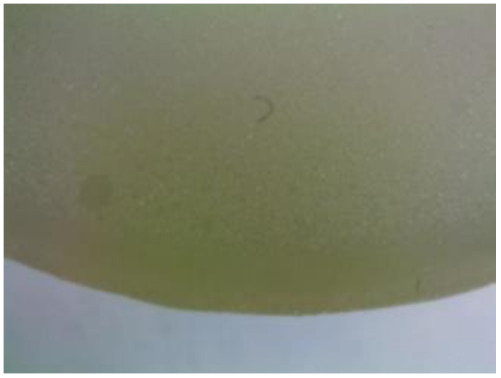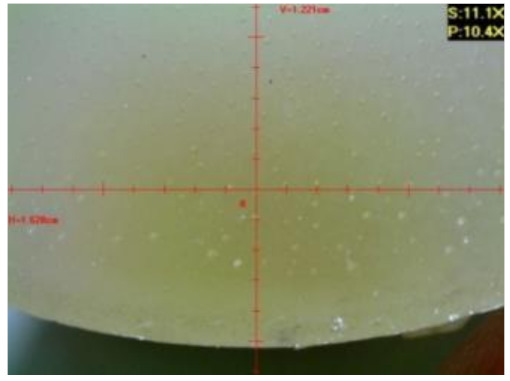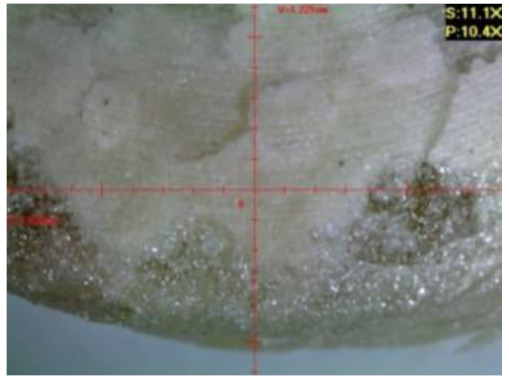# The Impact of the Acidic Environment on the Mechanical Properties of Epoxy Compounds in Different Conditions

**DOI:** 10.3390/polym12122957

**Published:** 2020-12-10

**Authors:** Anna Rudawska

**Affiliations:** Lublin University of Technology, Faculty of Mechanical Engineering, Nadbystrzycka 36 Str, 20-618 Lublin, Poland; a.rudawska@pollub.pl; Tel.: +48-81-53-84-232

**Keywords:** epoxy compounds, exposure time, aqueous solution, acetic acid, mechanical properties

## Abstract

The aim of this work was to determine the impact of the acidic environment on the mechanical properties of two epoxy compounds in different conditions. The samples were made from the epoxy compounds composed of the epoxy resin (based on Bisphenol A), triethylenetetramine curing agent (unmodified compound), and calcium carbonate (CaCO_3_) (modified compound). The epoxy compound samples were seasoned for the following period of time (i.e., one week, one month, and three months). The environment was tap water and the acidic environment had three different concentrations of acetic acid (3%, 6%, and 9%). Strength tests of the epoxy compound samples were carried out in accordance with the ISO 604 standard. In the case of the modified composition, it is noted that the samples immersed in tap water were characterized by a higher strength than in acidic environments. A similar tendency was observed for unmodified compositions, although the differences were smaller than for the modified compositions. It was also noticed that the increase in the pH of the acidic solution in many analyzed cases contributed to the decrease in mechanical properties, although the immersion time in the acidic solution is important.

## 1. Introduction

A wide variety of epoxy resins have become the most popular polymers since the mid 20th century [1,2]. These are used in different industries, especially in electronics and electrical engineering, automotive, aviation, and construction engineering to bond metals, polymers, glass, and wood [3,4,5,6,7]. Epoxy compounds can be employed in a wide range of possible applications.

The important and typical characteristics for the epoxy compounds are high mechanical resistance, good adhesion to different types of materials, and resistance to weather and environmental effects as well as the possibility of modification to obtain specific properties [1,8,9,10,11]. All these characteristics have a positive impact on the superiority of epoxy compounds over the other ones [12,13,14]. Due to their very good resistance, epoxy compounds are the leading competitors of the most popular material bonding methods such as welding, riveting, or soldering [3]. However, it must be pointed out that the environmental factors including temperature, humidity, and environmental aggressiveness, among others, have a tremendous impact on the strength of the epoxy compounds [15,16,17,18,19,20,21,22,23,24,25,26] as well as in an acidic environment among the aqueous environments. Various elements made of epoxy polymer composites (e.g., in chemical processes or building and assembly process related to the cleaning of these elements) can be exposed to the acidic environment.

Works dealing with the analysis of the degradation process of epoxy compositions in various aqueous environments have previously been published [19,21,24,27,28,29,30,31,32]. In the work by Rudawska and Brunella [33], the effect of operating factors such as seasoning in water solution, containing iron sulfate, and the time of seasoning was investigated. Based on the obtained results, it has been found that too high an iron sulfate content in water has a negative effect on the selected mechanical properties of the epoxy compound. In other works by Rudawska et al. [34], Rudawska [35], and Miturska et al. [36], the seasoning time was also one of the factors analyzed during the aging research. It can be noticed that both the temperature and time of seasoning have an impact on the mechanical properties of the epoxy compounds. It can also be observed that the compressive strength of the epoxy compound samples seasoned at ambient temperature increased as the seasoning time increased. Based on the results [36], it can be seen that the type of filler affects the strength of the tested compositions over the exposure time. In the case of montmorillonite, the strength increases, but as far as the addition of carbon and chalk is concerned, the strength decreased over the exposure time for the tested epoxy resin compounds.

The processes occurring in epoxy compounds, epoxy compositions during aging, or interaction in various environments, based on the environmental factors, are also affected by the type of epoxy compound itself as well as the type of additive/modifying agent. One such additive is CaCO_3_, and the issues related to the modification of epoxy resins with this additive are presented in several papers [37,38,39,40,41]. CaCO_3_, as a modifying agent, is considered to be a suitable substitute for rubbery elastomer in the epoxy resin matrix due to the fact that it is characterized by high rigidity and may influence the behavior of the rubber elastomer [42]. Li et al. presented the study on the mechanical properties of the epoxy composite modified with nano-sized CaCO_3_ particles [42]. Sosiati et al. investigated the effect of the CaCO_3_ particle size and the ratio of kenaf to CaCO_3_ content on the impact strength of the hybrid composite based on the epoxy resin [43]. The predictability of the impact strength of the CaCO_3_ reinforced epoxy resin composite based on the values of the input concentration and sustained stress at impact was tested by Nwoye et al. [44]. Abd presented the study of the effect of different weight ratios of CaCO_3_ particles and different polyvinyl chloride (PVC) content on the mechanical and electrical properties of epoxy composition [45]. The effect of nano-CaCO_3_ content on the mechanical and thermal properties of epoxy/nano-CaCO_3_ composites was tested by He et al. [46]. He et al. also investigated the moisture absorption and diffusion of epoxy-based nanocomposites containing nano-CaCO_3_ [47]. The effect of CaCO_3_ and Al_2_O_3_ fillers on the mechanical properties of glass/epoxy composites was studied by Sai Sravani et al. [48]. Mechanical wear and thermal properties of the epoxy composite reinforced with CaCO_3_ powder was investigated by Al-Zubaidi et al. [49] and the results showed that the addition of CaCO_3_ powder to the epoxy resin matrix produced a harder, less ductile, and better heat insulator product.

Some authors (e.g., Li et al. [42]) have shown that the addition of CaCO_3_ can have a negative impact on the mechanical performance of polymers. Sai Sravani et al. [48] also indicated that there was a decrease in tensile and flexural strength with the addition of filler material. However, He et al. [46] demonstrated that even small contents (2–6 wt.%) of nano-CaCO_3_ in the epoxy matrix (in cured state) can increase the thermal stability and mechanical properties of the modified epoxy material. On the other hand, with regard to the exploitation process in the aquatic environment, He et al. [47] presented results that the negative effect of moisture on the flexural property of unmodified epoxy can be reduced by adding nano-CaCO_3_ particles. The effect of nano-CaCO_3_ on the mechanical properties was found to be positive, which is promising for the application of modified epoxy resin in high-humidity environments. However, different environments and exposure time can affect the properties of the modified epoxy compositions.

The analysis of the compressive strength in the work answers the question of what load should be applied to damage the structure of the insulating material. The higher the compressive strength value, the greater the load on the material can be. This relationship is widely used in engineering because it allows the application of a specific material (e.g., construction) where the load acting on building structures is mainly snow, wind, earth, and other materials including water (also acid environments).

The main focus of the present article was on examining the impact of tap water and acidic environments of three different concentrations on the resistance of two types of epoxy compounds based on the epoxy resin (based on bisphenol A), taking into consideration different periods of exploitation. This article presents the research concerning the impact of one of the exposure variants, which was an acidic environment in the form of an aqueous solution of acetic acid, on the mechanical properties of two epoxy compounds: a modified calcium carbonate powder (CaCO_3_) and an unmodified one.

## 2. Materials and Methods

### 2.1. Epoxy Compounds

Two types of epoxy compounds containing epoxy resin and curing agent (in the case of the unmodified compound) as well as CaCO_3_ powder (in the case of the modified compound) mixed in appropriate weight proportions were used to make test samples. The quantitative proportion of the constituents used is presented in Table 1. The appropriate proportions of the constituents allowed us to obtain epoxy compounds of the desired characteristics.

Epoxy resin is a styrene-modified epoxy resin based on bisphenol A (Epidian 53 trade name, CIECH Resins, Nowa Sarzyna, Poland). The curing agent is an amine curing agent (triethylenetetramine curing agent, Z-1 trade name, CIECH Resins, Nowa Sarzyna, Poland). The properties of the used epoxy resin and curing agent were presented in [33,36]. Strength parameters of the used epoxy resin with the addition of the triethylenetetramine curing agent after being cured for seven days at ambient temperature are presented in Table 2.

CaCO_3_, in the form of a powder (micro-filler) with a molecular weight of 100.09 g/mol, which is an inorganic chemical compound-a salt of carbonic acid and calcium, was used as a modifier. CaCO_3_ is commercialized by Carl Roth GmbH + Co KG, Karlsruhe, Germany, with the trade name E170. According to the literature [8,45], the amount of CaCO_3_ as a modifying additive for resins or polymer composites is in the range of 2–8 parts by weight. Yang et al. [8] underlined that the mechanical properties of the composites were enhanced by increasing the amount of CaCO_3_ added, but decreased when the filler content reached 2%. He at al. [46] mentioned that performance of polymeric materials can be improved by introducing some small amounts of filler <5 wt.%. The addition of 2 g of CaCO_3_ per 100 g of epoxy resin was used in the study.

### 2.2. Samples

**Figure 1:**
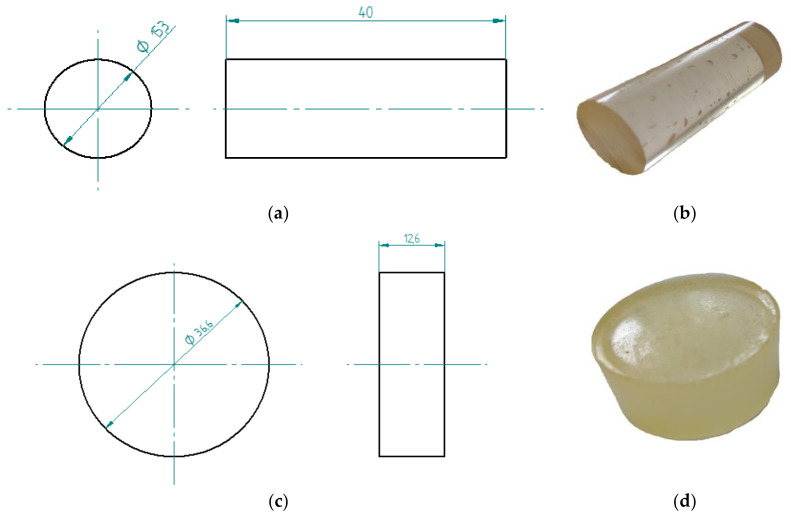
Epoxy compound samples: (**a**) the sample’s scheme and dimensions (in mm) for strength tests, (**b**) a view of the sample made of Epidian 53/Z-1 for strength tests, (**c**) the sample’s scheme and dimensions (in mm) for microscopic tests, (**d**) a view of the sample made of Epidian 53/Z-1/CaCO_3_ for microscopic tests.

The tests included two types of the epoxy compound samples whose characteristics were presented in Table 1: (i) samples for the strength tests and (ii) samples for the microscopic tests. The former (i) were cylinder-shaped and made on the 10 mL-forms. The scheme of the epoxy compound samples was presented in Figure 1a,b.

The samples were made of two types of epoxy compound. Seventy-two samples were made of each type of epoxy compound (six samples × three exposure times × four environments variants, Table 3) for the strength test. The total number of the prepared cylindrical samples was 144. The scheme of the epoxy compound samples for the microscopic tests (ii) is presented in Figure 1c,d. These samples were prepared in a silicone form containing several nests with the diameter of 37 ± 0.2 mm and of height of h = 13 ± 0.2 mm. For microscopic tests, 12 round samples were made of each epoxy compound type. These were exposed to different exploitation environments for three different seasoning periods shown in Table 3.

### 2.3. Technology of Epoxy Compounds Sample Preparation

The process of the test samples’ preparation included a few phases: preparing forms to make the samples, preparing an epoxy compound, filling the forms with an epoxy compound, curing the epoxy compounds, taking the samples out of the forms, and mechanical processing of the samples. Preparation of the epoxy compound was preceded with the preparation of cylindrical forms. It included spraying the inner surface of the forms with a silicone Polsiform release agent (the producer: Polish Silicones, Nowa Sarzyna, Poland), which is a transparent and scentless preparation used, among others, in rubber and polymer processing. Covering the forms with the aforementioned agent enabled us to create a thin layer of the silicone oil that made it possible to take the cured epoxy compound samples out later on.

The next phase was to prepare the epoxy compound. Taking into consideration the appropriate weight proportions, the particular ingredients of the specific epoxy compound (i.e., epoxy resin (Epidian 53), triethylenetetramine curing agent (Z-1), and CaCO_3_) were measured with the use of electronic scales. The weight proportions used to prepare the specific epoxy compounds are presented in Table 1. The ingredients were mixed mechanically at a mixing station with the use of a horseshoe mixer. The mixing speed was 460 rpm and the mixing time was 2 min. Efforts were made so that no air bubbles are created within the epoxy compound mass. After being mixed, the epoxy compound was also subjected to a 2-min degassing process at the special station equipped with a container and a vacuum pump. In the case of the epoxy compound with CaCO_3_, this modifier was added to the epoxy resin first. The curing agent was added to the ingredients mixed beforehand. The mixing time of the three-ingredient compound (modified) was 2 min, which allowed us to distribute the CaCO_3_ in the epoxy compound evenly.

The conditions of preparing and curing the samples for both the strength and the microscopic tests were as follows: air temperature of 20 ± 2 °C and relative air humidity of 25 ± 2%. Time of curing in the forms was seven days. After that, the cured epoxy compound samples were taken out of the forms. They were then subject to mechanical processing in order to equalize their length.

The epoxy compound (modified and unmodified) samples prepared in such a way were exposed to environmental factors (specific environment and seasoning time) (Table 3). The unaged samples were not exposed to any exploitation factors. The storage conditions for the unaged samples were the same as for the curing process. Thanks to that, it was possible to compare the impact of time and the exploitation environment on the strength of the epoxy compounds.

The operating environments presented in Table 3 were prepared (except for tap water) and placed in glass vessels. Samples of the epoxy compositions were immersed in these media in such a way that the aqueous solutions fully covered the samples. The prevailing ambient conditions in which the glass containers with rotors and samples were immersed, were as follows: temperature of 20 ± 2 °C and relative air humidity of 25 ± 2%. The measurements of the pH of water environments were made with the use of pH indicators (indicator papers pH 0–12, Stanlab, Lublin, Poland). The tap water showed a pH equal to 7, and the remaining aquatic environments showed the following values: RKO3% pH = 4, RKO6% pH = 3, and RKO9% pH = 3. The tap water used in the experiments was bicarbonate–calcium–magnesium water, and its characteristics are presented in Table 4. The content of the calcium carbonate amounted to 381 mg/L [33].

After the assumed seasoning time in the acidic environments (Table 3), the epoxy compound samples were conditioned prior to further testing. The conditioning time was 48 h, temperature was 20 ± 2 °C, and the relative humidity was 25 ± 2%. After conditioning the epoxy compounds, samples were subjected to strength tests as well as to the microscope tests.

### 2.4. Compression Tests

Compression tests of the epoxy compound samples were carried out with accordance to the norm ISO 604 on the testing machine Zwick/Roell Z150 (ZwickRoell GmbH&Co. KG, Ulm, Germany). The parameters used during the strength tests of the epoxy compound samples were the following: initial force of 10 N, compression modulus speed of 2 mm/min, crosshead speed of 10 mm/min, force cut-off point of 80% Fmax, and maximum unit shortening of 10%. Six samples were tested in each run of the strength tests.

### 2.5. Microscopic Tests

Microscopic tests were carried out with the use of the DiGi ViTiny UM06 microscope (ViTiny, Kaohsiung, Taiwan). The main objective of the microscopic tests was to observe the surface of the samples in order to determine the signs of influence of the exploitation environments, the exposure time, and presumable material discontinuity (e.g., cracks or air bubbles).

## 3. Results

### 3.1. Unmodified Epoxy Compounds

#### 3.1.1. Compression Test Results

**Figure 2:**
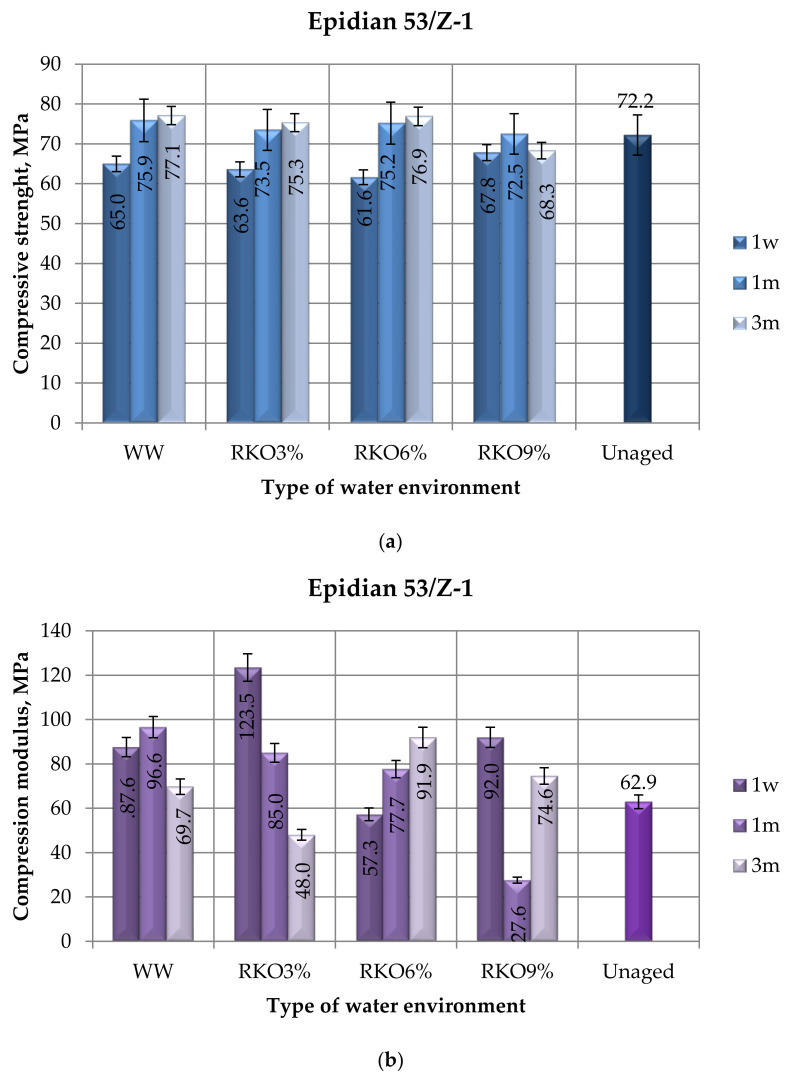

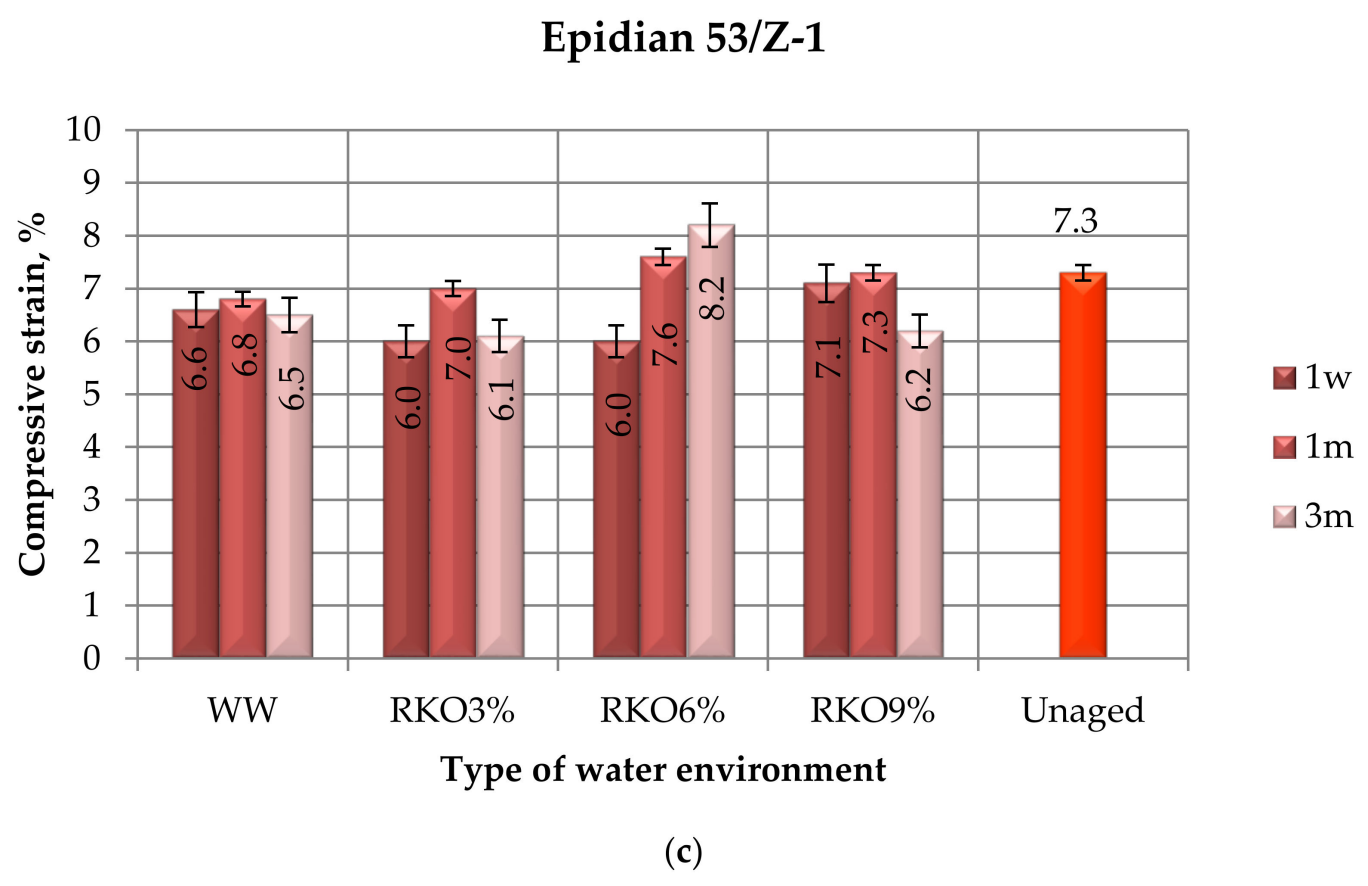
Results of the compression tests of the unmodified epoxy compound (Epidian 53/Z-1) seasoned in four water environments for the following periods of time: one week, one month, and three months: (**a**) compressive strength, (**b**) compression modulus, (**c**) compressive strain.

The results of the compression tests (compressive strength, compression modulus, and compressive strain) of the unmodified epoxy compound (Epidian 53/Z-1) seasoned in four water environments (Table 3) for the following periods of time: one week (1 w), one month (1 m), and three months (3 m) are presented in Figure 2. The results of the compression tests of the unaged samples (not exposed to the exploitation conditions) of the unmodified epoxy compound are also presented in Figure 2.

When comparing the compressive strength of the unmodified compound’s samples with the same seasoning time, but different water environments (Figure 2a), it was observed that the samples’ strength was at a similar level. Taking into consideration the seasoning time in particular water environments, it may be stated that the strength increases with the seasoning time (apart from one case of seasoning in the 9% aqueous acetic acid solution). Some minimal negative effect of the epoxy compound samples’ seasoning in an acidic environment was observed though. The compressive strength of the unmodified epoxy compound is insignificantly lower in the water acidic environments, regardless of the seasoning time. In the case of the epoxy compound samples seasoned for both one month and three months, the compressive strength was higher for the samples seasoned in tap water. Only in one case—seasoning for one week—was the epoxy compound’s strength after seasoning with the highest concentration of the aqueous solution of acetic acid (9%) slightly higher than the strength of the epoxy compound seasoned for the same period of time, but in tap water. Comparing the results of the compressive strength of the samples subjected to seasoning at different times and in a different water environment with the unaged samples, it is noted that in each water environment, in the case of seasoning for one week, the samples of the unmodified epoxy compounds were lower than the unaged samples (72.2 MPa). In the remaining analyzed cases, the compressive strength of the unaged samples was lower (except for one case).

The comparison of the results presented in Figure 2b, which was related with the comparison of the compression modulus values for the analyzed seasoning variants, allowed us to make the following observations. In relation to the seasoning period of one week, the highest compression modulus was observed for the epoxy compound’s samples that were seasoned in the 3% acetic acid aqueous solution. In the case of the samples seasoned in this environment, the average compression modulus was 123.5 Mpa and was over half as high as for the samples of the epoxy compound seasoned in the 6% acetic acid aqueous solution, which showed the lowest compression modulus of all types of seasoning environments. Considering the seasoning period of one month in the water environments, the highest compression modulus was observed for the epoxy compound’s samples that were seasoned in tap water. This was 96.9 Mpa and over 70% higher than for the samples of the epoxy compound seasoned in the 9% acetic acid aqueous solution, which showed the lowest compression modulus (27.6 Mpa). It was also observed that with the increase in the acetic acid concentration in the acidic solution during the one month seasoning, the value of the compression modulus decreased. In the case of the three month seasoning period, it could be observed that the value of the compression modulus for the analyzed samples may be considerably differentiated. The highest compression modulus (91.6 Mpa) was observed for the epoxy compound samples that were seasoned in the 6% acetic acid aqueous solution. The compression modulus for the samples seasoned in the 9% acetic acid aqueous solution was almost 20% lower. The lowest compression modulus was 36.5 Mpa and was observed for the samples seasoned in tap water. In most of the analyzed cases (except for three variants of samples for different environments and different seasoning times–RKO3%/3m, RKO6%/1w, RKO9%/1m), the compressive modulus of the unaged samples was lower than the samples subjected to the environmental factors.

In the shortest seasoning period of time (one week), the highest compressive strain was observed for the samples seasoned in the 9% acetic acid solution and was 7.1% (Figure 2c). The very same strain was also observed for the samples seasoned in the 3% and 6% acetic acid solution and was 15% lower than the highest compressive strain, which was 7.1%. The compressive strain of the epoxy compound samples that were seasoned for one month ranged from 6.8% to 7.6%, whereas the highest value was observed for the samples seasoned in the 6% acetic acid aqueous solution and the lowest value was for the samples seasoned in tap water (WW). For the 3-month seasoning period, the highest compressive strain was observed for the samples seasoned in the 6% acetic acid solution and was 8.2%. The lowest strain was observed for the epoxy compound samples that were seasoned in the 3% acetic acid aqueous solution. It was observed that the samples seasoned for one month in the majority of water environments had the highest compressive strain values (except for the 6% acetic acid aqueous solution). With regard to the unaged samples, it can be noticed that, apart from three cases of seasoning (two of them concern the RKO6% environment, one month and three months), the compressive strain of the unaged samples was greater than those of the samples seasoned in the tested environment, consisting mainly of all acidic environments. The obtained results with respect to the adopted aging time in the applied environments showed a certain negative trend in the form of reducing the compressive strain value of unmodified epoxy compounds.

#### 3.1.2. Microscopic Images and Failure Form of Unmodified Epoxy Compound Samples

The microscopic test images conducted on the samples of the unmodified epoxy compound seasoned in exploitation environments are presented in Table 5. Designations of seasoning time and exploitation environments are presented in Table 3.

The microscopic tests disclosed the tested epoxy compounds’ microstructure development, structure changes, and defects that are not distinguishable to the unaided eye. It was observed that as the seasoning time passed by, the samples seasoned in all the exploitation environments (i.e., tap water and the 3%, 6%, and 9% acetic acid solutions) changed their color (Table 5). In addition, when observing the samples under magnification, it was noted that their structure was changed; this became rough and the air bubbles appeared. CaCO_3_ can react with acetic acid in some cases and thus would produce a porous structure. The most visible impact on the unmodified epoxy compound’s structure was exerted by the 3% acetic acid solution, whose samples were seasoned for three months. The sample’s surface was much rougher, the air bubbles that appeared on the surface were much bigger than in the case of the samples seasoned for a shorter period of time. The 6% acetic acid had the same impact on the samples seasoned for one month.

**Figure 3:**
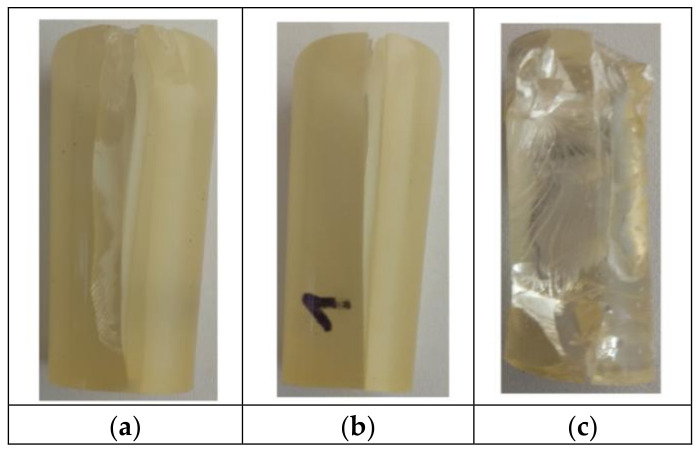
Forms of failure of the compressed modified epoxy compound samples: (**a**) cracking, (**b**) buckling and cracking, and unmodified epoxy compound’s sample; (**c**) spalling.

The samples of epoxy compounds were subject to the ideal axial and centric compression. During the compression of the epoxy compound samples, the following deformations were observed: buckling, spalling, and cracking. Several samples were completely damaged. Figure 3 shows the exemplary forms of failure of the compressed epoxy compound samples. The photos of the samples shown in Figure 3 were taken by a digital camera.

### 3.2. Modified Epoxy Compounds

#### 3.2.1. Compression Test Results

**Figure 4:**
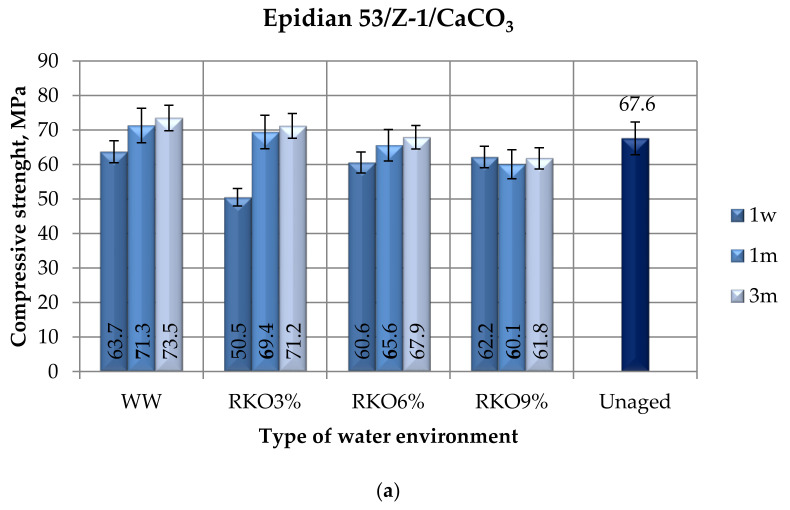

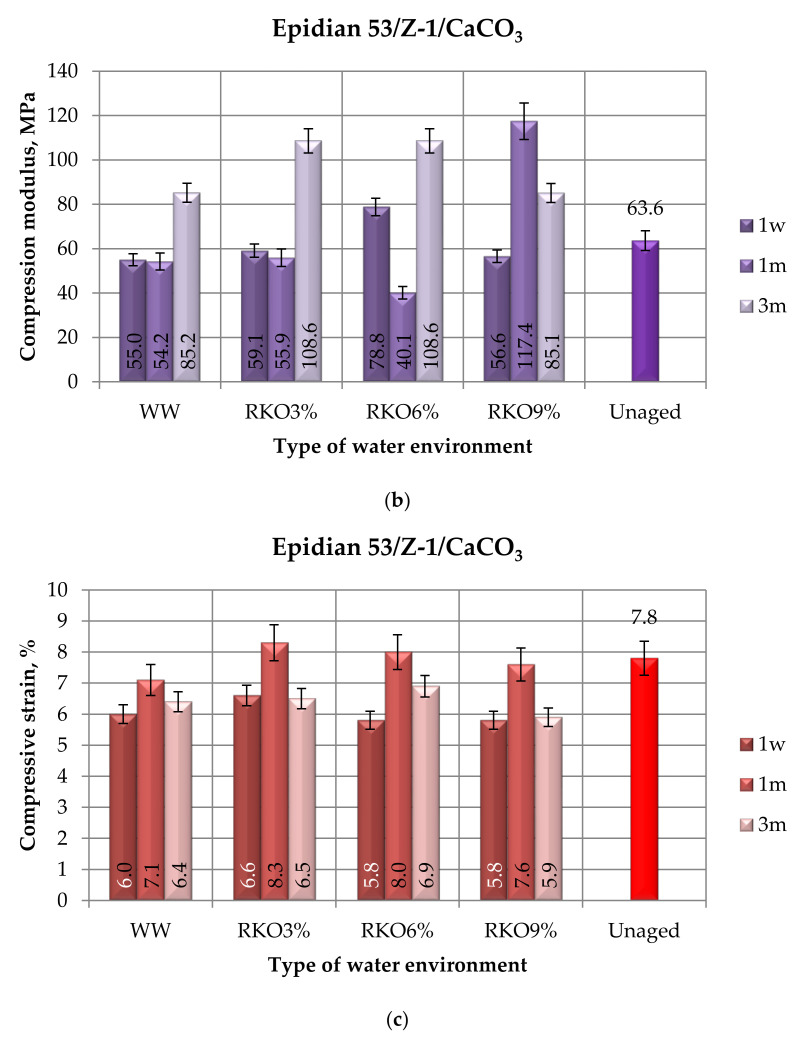
Results of the compression tests of the modified epoxy compound (Epidian 53/Z-1/CaCO_3_) seasoned in four water environments for the following periods of time: one week, one month, and three months: (**a**) compressive strength, (**b**) compression modulus, (**c**) compressive strain.

The results of the compression tests (compressive strength, compression modulus, and compressive strain) of the modified epoxy compound (Epidian 53/Z-1/CaCO_3_) seasoned in four water environments (Table 3) for the following periods of time: one week (1 w), one month (1 m), and three months (3 m) are presented in Figure 4. The results of the compression tests of the unaged samples (not exposed to the exploitation conditions) of the modified epoxy compound are also presented in Figure 4.

Based on the results shown in Figure 4a, taking into consideration the seasoning time of the modified epoxy compound, the following dependences may be observed. Taking into consideration the one week seasoning period, the samples seasoned in tap water had the highest compressive strength (Figure 4a). However, it was observed that the difference in the compressive strength value between the samples seasoned in tap water and both the 6% and 9% acetic acid solutions were not significant. The lowest compressive strength was for the samples seasoned in the 3% acetic acid aqueous solution. Their strength value was almost 20% lower than the highest one. The results obtained for the samples seasoned for one month showed that the highest average strength (71.1 MPa) was obtained by those seasoned in tap water. This environment type appeared to have almost 3% higher impact on the epoxy compound’s strength than the 3% acetic acid solution and was 16% higher than the 9% acetic acid solution. It was also observed that with the increase in the acetic acid’s concentration in the acidic solution, the value of the compressive strength decreased. The compressive strength of the modified epoxy compound’s samples that were seasoned for three months in water environments ranged from 61.8 MPa to 73.5 MPa. The highest compressive strength was observed for the samples seasoned in tap water, whereas the lowest was for the samples seasoned in the 9% acetic acid aqueous solution. The highest compressive strength was observed for the samples seasoned for three months, regardless of the water environment type. The exception was the 9% acetic acid aqueous solution, for which the strength values were very similar, regardless of the seasoning time.

Comparing the results of the compressive strength of the samples subjected to seasoning at different times and in a different water environment with the unaged samples, it is noted that in each water environment, in the case of seasoning for one week, the samples of the unmodified epoxy compounds were lower than the unaged samples. In the case of two acidic environments (aqueous solution of acetic acid 6% and aqueous solution of acetic acid 9%), the strength of the refrain samples was higher than that of the samples of the compositions seasoned in these environments, regardless of the seasoning time.

With respect to the results of the compression modulus (Figure 4b), the following dependences were observed. In relation to the seasoning period of one week, the highest compression modulus (78.8 MPa) was observed for the epoxy compound samples that were seasoned in the 6% acetic acid aqueous solution. The lowest compression modulus was 55 MPa and was observed for the samples seasoned in tap water. The difference was 30%. For the one month seasoning period, the highest compression modulus (117.4 MPa) was observed for the epoxy compound samples that were seasoned in the 9% acetic acid aqueous solution. This value deviated significantly from the other sample results. The lowest compression modulus value was obtained by the samples seasoned in the 6% acetic acid solution (40.1 MPa) and it was over 65% lower than the highest value for the same seasoning time. For the three month seasoning period, the highest compression modulus value (108.6 MPa) was observed for the epoxy compound samples that were seasoned in both 3% and 6% acetic acid aqueous solution. The compression modulus for the samples that were seasoned in the 9% acetic acid aqueous solution was about 22% lower. It was observed that the three month seasoning time (except for one variant) had a positive impact on the increase in the value of the compression modulus in comparison to other seasoning time variants, regardless of the seasoning environment.

The results of the compressive strain of the epoxy compound 53/Z-1/CaCO_3_ seasoned in four water environments for the following periods of time: one week, one month, and three months (Figure 4c) allowed us to present the following observations. In the case of the one week seasoning time, the highest compressive strain was 6.6% and it was observed for the samples seasoned in the 3% acetic acid solution. The samples seasoned in tap water showed a slightly lower compressive strain value, which was 6%. The lowest compressive strain was observed for the samples seasoned in both 6% and 9% acetic acid solution and was 5.8%. When analyzing the results obtained after the one month seasoning, it may be observed that the average compressive strain of the epoxy compound samples ranged from 7.1% to 8.3%. The highest compressive strain was observed for the samples seasoned in the 3% acetic acid solution, whereas the lowest was for the samples seasoned in tap water. For the three month seasoning period, the highest compressive strain was observed for the samples seasoned in the 6% acetic acid solution and was 6.9%.

The lowest compressive strain, in turn, was observed for the samples seasoned in the 9% acetic acid solution and was 5.9%. In conclusion, however, it can be noticed that the differences in the obtained results were small.

#### 3.2.2. Microscopic Images of Modified Epoxy Compound Samples

The photographs presenting the microscopic images of the modified epoxy compound Epidian 53/Z-1/CaCO_3_ samples are presented in Table 6.

Like in the case of the samples made from the unmodified epoxy compound (Epidian 53/Z-1), the samples of modified epoxy compound (Epidian 53/Z-1/CaCO_3_) seasoned in the exploitation environments changed color as time passed by. A special change was observed for the sample seasoned for three months in the 6% acetic acid solution and 9% acetic acid solution (Table 6). The appearance of these samples was significantly changed. The samples of the modified epoxy compound samples seasoned in the 9% acetic acid aqueous solution were covered with a white deposit, which together with air bubbles, led to the spalling of the epoxy compound. Fine air bubbles were found on the surface of almost all epoxy compound samples. This resulted in the increase in the surface roughness of the samples. The air bubbles were especially visible on the sample seasoned for one month in the 3% acetic acid solution (Table 6).

## 4. Discussion

It must be pointed out that numerous factors may have had an impact on the test results of the mechanical properties of the compounds. They may include, among others, the method of the epoxy compound’s preparation, temperature, and humidity in which the compounds were being prepared and cured as well as the seasoning time and type of exploitation environment. In a previous work, Rudawska showed that both temperature and time of seasoning had an impact on the mechanical properties of epoxy compounds [35]. From the perspective of eight months, these changes were relatively minor for the samples seasoned at ambient temperature. In [33], it was underlined that too high an iron sulfate content in water had a negative effect on the mechanical properties of the epoxy compound samples. Miturska et al. concluded that the type of filler affects the strength of the tested compositions over the seasoning time [36]. With regard to the immersion of the samples in acidic solutions, it has been shown that unmodified and modified epoxy compounds have different mechanical properties and it is difficult to unequivocally determine the impact of the acidic environments on these properties. Additionally, Banna et al. [51] studied the effects of two aqueous acid solutions on different resins and concluded that the resins presented different mechanical behavior under aggressive solutions. Amaro et al. [52] studied the flexural and low velocity impact response of a glass fiber/epoxy composite after immersion in HCl and H_2_SO_4_. It was shown that the flexural strength and flexural modulus were affected significantly by the acid solutions. Moreover, the exposure time was shown to be a determinant of the flexural properties. Moreover, Belloul et al. [53] presented the behavior of the glass fiber/unsaturated polyester (UP) composite with structural defects subjected to the attack of two corrosive solutions: H_2_SO_4_ and NaOH. The resistance and ductility losses revealed by the mechanical tests of the samples immersed in the two mediums of immersions are the result of a complex mechanism degradation implementing several phenomena such as the resin hydrolysis, the fiber corrosion, and the degradation of the fiber/matrix interface, in addition to the structural defects due to the implementation technique.

When considering the pH value, the water environments can be observed, and based on the obtained results, it was noticed that the increase in the pH of the acidic solution (for the RKO6% and RKO9% variants) in many analyzed cases contributed to the decrease in mechanical properties, although the immersion time in the acidic solution was important. Mahmoud and Tantawi [54] underlined that changes in the flexural strength, hardness, and Charpy impact resistance depends upon the type of acid and the period of immersion. Comparing the results of the compressive strength (Figure 2a and Figure 4a), it was observed, especially for the modified composition, that depending on the time of immersion in tap water with a pH value of 7, the compressive strength was higher than for the variant of the immersion of samples of this composition in a solution with a pH 4 value and pH 3 value. Slightly smaller differences were noticed for the unmodified composition (Figure 3), but the compressive strength of the samples of the unmodified composition immersed in the water environment with a pH 7 value than in the water environment with a pH 4 value and pH 3 value could also be indicated. Stamenovic et al. [55] investigated the effect of alkaline and acid solutions, and they presented that the alkaline solution decreased the tensile strength and this tendency increased with the pH value.

Regarding the modification of the epoxy compositions with CaCO_3_, it can be noted that the unaged samples of the unmodified epoxy compound (Epidian 53/Z-1) had a higher strength than the unaged samples of the modified epoxy compound (Epidian 53/Z-1/CaCO_3_). This conclusion can be applied to all environments (water and acid solutions) and times used in the experiments, although in some of the compared cases, the differences were not very significant. Li et al. underlined that micron-sized CaCO_3_ may have a negative impact on the mechanical performance of polymers [42]. Some researchers (e.g., Delozanne et al. [27], Calvez et al. [28], Bowditch [29], and others [7,16,23,24]) have reported that moisture in bulk epoxy compounds tends to migrate to the epoxy compound–substrate interface and decreases the adhesion force there. Sai Sravani et al. also indicated that there was a decrease in tensile and flexural strength with the addition of filler material [48]. In addition, the samples of the unmodified epoxy compound Epidian 53/Z-1 after being seasoned in tap water and the acetic acid solution of three different concentrations for the assumed period of time showed lower strength than the unaged samples. The samples of the modified epoxy compound Epidian 53/Z-1/CaCO_3_, however, showed a higher strength after seasoning. Higher values of the compressive strength after seasoning in different environments were observed for the modified epoxy compound 53/Z-1/CaCO_3_. The highest compressive strength was 15.2 MPa, where in the case of the unmodified epoxy compound Epidian 53/Z-1, the highest value was 9.6 MPa. The compressive strain at the compressive strength for both compounds was at a similar level. According to He et al., nano-CaCO_3_ particles have a large surface area and they may act as efficient barriers against moisture transport through the polymers [47]. The transport speed of moisture or vapors through the polymers is retarded because the impenetrable nanoparticles cause an increase in the path length for molecule diffusion through the polymer. He et al. also underlined that the increase in equilibrium moisture content observed with the increase in nano-CaCO_3_ content can be explained by the growth of void content and interphase layer content [47]. Moreover the negative effect of moisture on the flexural property of unmodified epoxy can be reduced by adding nano-CaCO_3_ particles. Among all the samples made from the unmodified epoxy compound Epidian 53/Z-1, the highest compressive strength was observed for the samples seasoned in the 9% acetic acid solution for three months. The lowest compressive strength, in turn, was observed for the samples seasoned for three months in tap water (1.5 MPa). In the case of the modified epoxy compound 53/Z-1/CaCO_3_, the samples seasoned for one month in the 6% acetic acid solution showed the highest compressive strength (15.2 MPa). The least strong samples, in turn, were those seasoned for one month in the 9% acetic acid solution (2.7 MPa).

The samples designed for the microscopic tests were also subjected to tests. After analyzing the microscopic images of the epoxy compounds, it was observed that after being seasoned in different exploitation environments and periods of time, all samples changed their color. One of the mechanisms of destruction of the surface layers of polymer composites during the exposure to water is a bubble formation, which at a further stage may cause degradation of the polymer composite. In the conducted research, the appearance of bubbles was noticed in the microscopic images in the case of a longer seasoning in water, which can be one of the causes of polymer degradation. The effect of bubble based degradation on the physical and structural properties of single wall carbon nanotube (SWCNT)/epoxy resin composite samples was investigated by Hashemi and Mousavi [56]. Additionally, Belloul et al. [53] underlined that the presence of the pores (of rather significant size) in the material after its elaboration influences the diffusion kinetics of the acid and alkaline solutions and contributes to the mechanical degradation of the material. Moreover, air bubbles appeared on the surface, which made it rougher. In some cases, the samples changed their look. The changed samples were covered with a white deposit that, together with air bubbles, led to the spalling of the epoxy compound. Such a change was observed for the samples seasoned for three months in the 6% and 9% acetic acid solution.

## 5. Conclusions

The aim of this work was to determine the impact of the acidic environment on the mechanical properties of two epoxy compounds in different conditions. The subjects of the present research were two types of epoxy compound made of epoxy resin based on bisphenol A, the triethylenetetramine curing agent, and a modifier in the form of powdered CaCO_3_. The tests concerned the mechanical properties (compressive strength, compression modulus, and compressive strain) of the epoxy compounds that were subject to seasoning for different periods of time (one week, one month, three months) in various exploitation environments (tap water, 3%, 6%, and 9% acetic acid aqueous solutions). The obtained results showed the following:

No significant effect of exposure time in selected operating environments on the compressive strength of epoxy compounds was observed.

(i) In many cases, the unaged samples (not exposed to any water environment) of the unmodified epoxy compound had an insignificant higher compressive strength than the unaged samples of the modified epoxy compound, regardless of the seasoning variant, although there were different differences depending on the environment and immersion time.
(ii) In the case of the modified composition, it was noted that the samples immersed in tap water were characterized by a higher strength than in acidic environments. A similar tendency was observed for the unmodified compositions, although the differences were smaller than for the modified compositions.
(iii) The increase in the pH of the acidic solution in many analyzed cases contributed to the decrease (to a slight but noticeable degree) in the mechanical properties, although the immersion time in the acidic solution is important.

It can be assumed that during the exploitation in acidic environments, some kind of chemical reaction is triggered between the modifier and the acetic acid aqueous solution in the epoxy compound modified with CaCO_3_.

## Figures and Tables

**Table 1:** The quantitative proportion of the epoxy compounds.

Compound Type	Epoxy Resin	Curing Agent	Modifying Additive	Quantitative Proportion	Designation
Unmodified epoxy compound	Epidian 53	amine curing agent (Z-1)	-	100:10	Epidian 53/Z-1
Modified epoxy compound			CaCO_3_	100:10:2	Epidian 53/Z-1/CaCO_3_

**Table 2:** Strength parameters of the epoxy resin Epidian 53 with the addition of the Z-1 curing agent (mixed with a stoichiometric ratio of 100:10) after being cured for seven days at ambient temperature [50].

Tested Parameters	Values
Breaking stresses, MPa	40–60
Bending strength, MPa	80–100
Compressive strength, MPa	70–90

**Table 3:** Exploitation environments and times of the samples of the epoxy compounds: unmodified (Epidian 53/Z-1) and modified (Epidian 53/Z-1/CaCO_3_) together with their designations.

Environment	Time	Designation
Tap water (WW)	1 week	WW/1w
	1 month	WW/1m
	3 months	WW/3m
Aqueous solution of acetic acid 3% (RKO3%)	1 week	RKO3%/1w
	1 month	RKO3%/1m
	3 months	RKO3%/3m
Aqueous solution of acetic acid 6% (RKO6%)	1 week	RKO6%/1w
	1 month	RKO6%/1m
	3 months	RKO6%/3m
Aqueous solution of acetic acid 9% (RKO9%)	1 week	RKO9%/1w
	1 month	RKO9%/1m
	3 months	RKO9%/3m

**Table 4:** Selected physical and chemical parameters of tap water (presented in [33]).

Parameter	Unit	Content/Value
Chlorides	mg/L	29.4
Fluorides	mg/L	<0.4
Magnesium	mg/L	23.0
Sulfates	mg/L	36.4
Sodium	mg/L	9.2
Iron	μg/L	46
Calcium	mg/L	98
Hardness	mval/L	381

**Table 5:** Microscopic images of the unmodified epoxy compound seasoned in exploitation environments (10× magnification).

Seasoning Time		
1 week	1 month	3 months
**Environment–tap water**		
WW/1w	WW/1m	WW/3m
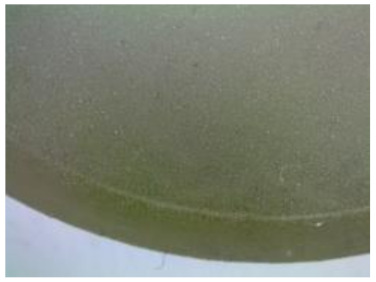	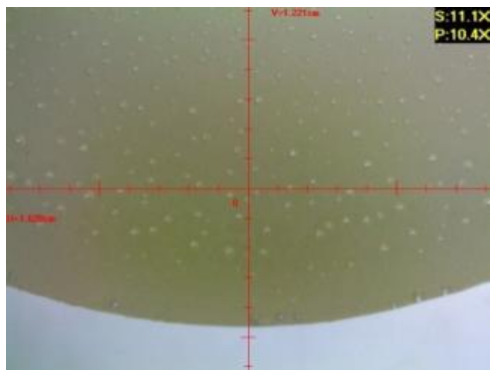	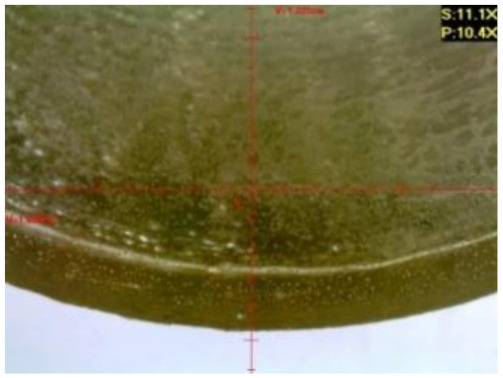
**Environment–aqueous solution of acetic acid 3%**		
RKO3%/1w	RKO3%/1m	RKO3%/3m
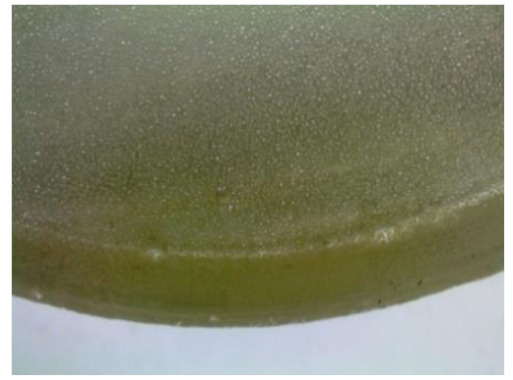	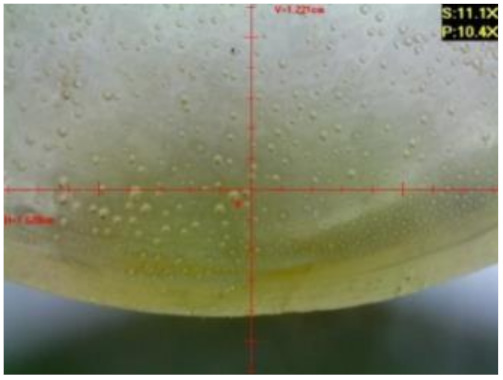	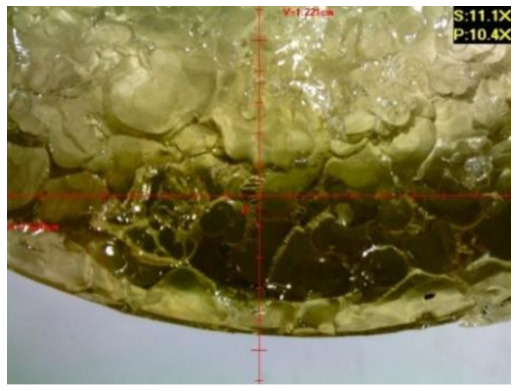
**Environment–aqueous solution of acetic acid 6%**		
RKO6%/1w	RKO6%/1m	RKO6%/3m
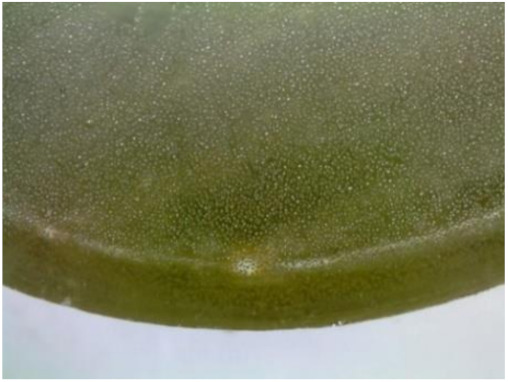	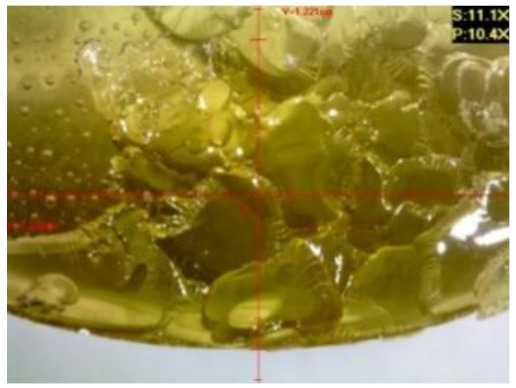	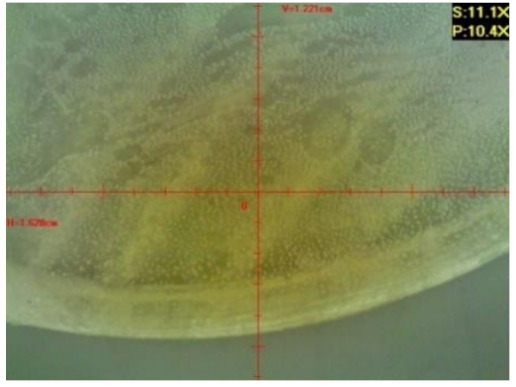
**Environment–aqueous solution of acetic acid 9%**		
RKO9%/1w	RKO9%/1m	RKO9%/3m
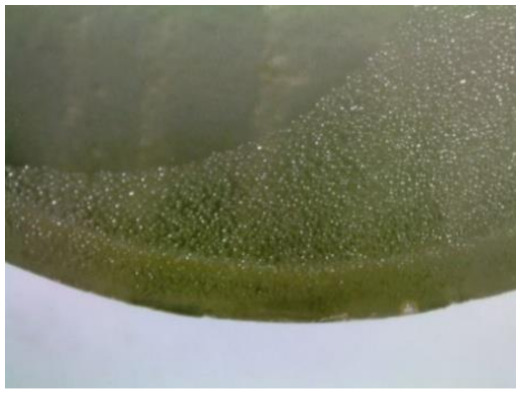	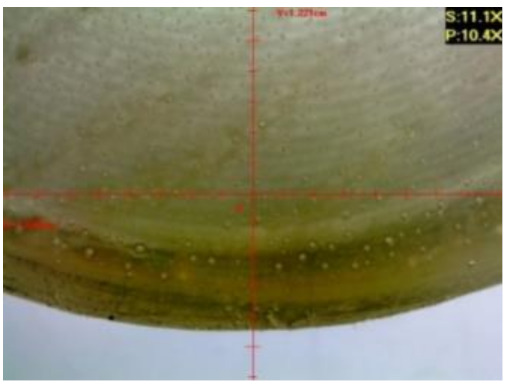	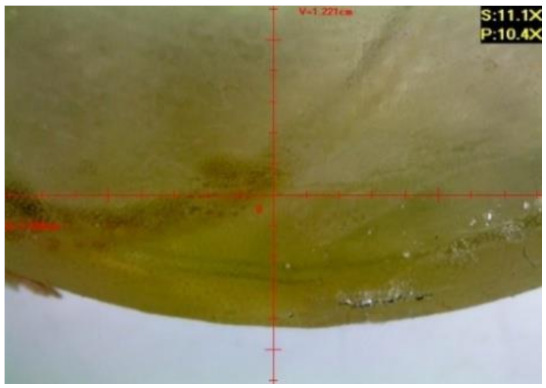

**Table 6:** Microscopic images of the modified epoxy compound seasoned in exploitation environments (10× magnification).

Seasoning Time		
1 week	1 month	3 months
**Environment–tap water**		
WW/1w	WW/1m	WW/3m
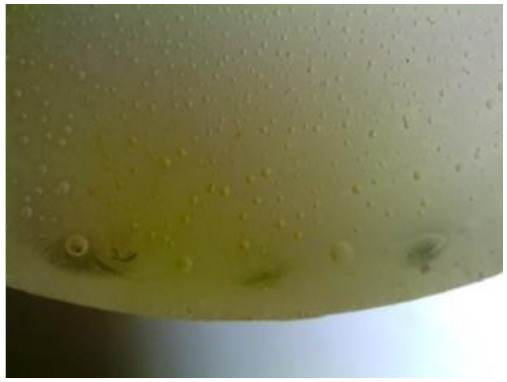	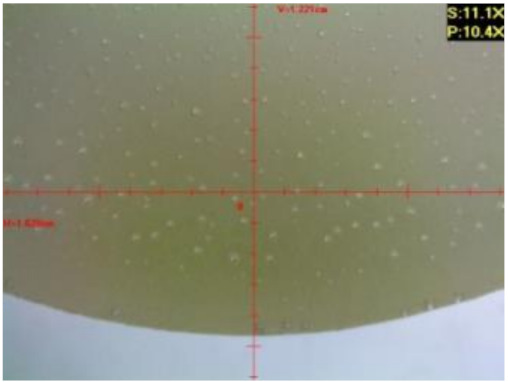	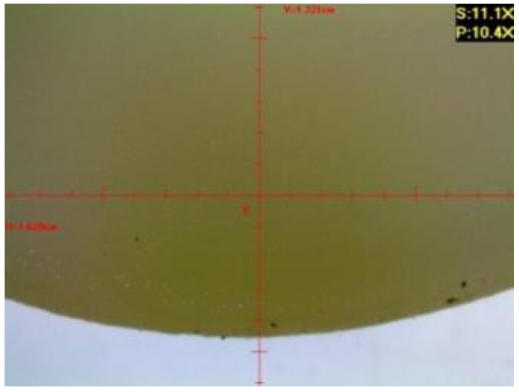
**Environment–aqueous solution of acetic acid 3%**		
RKO3%/1w	RKO3%/1m	RKO3%/3m
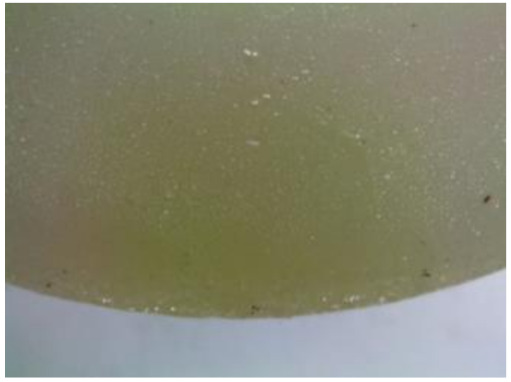	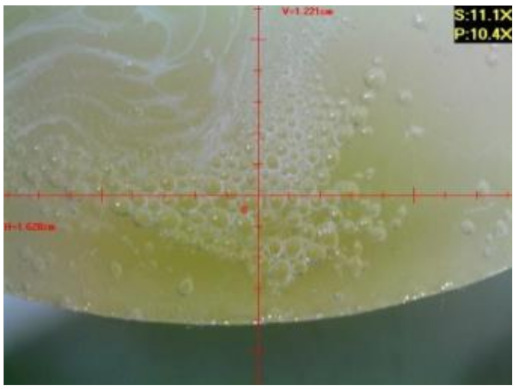	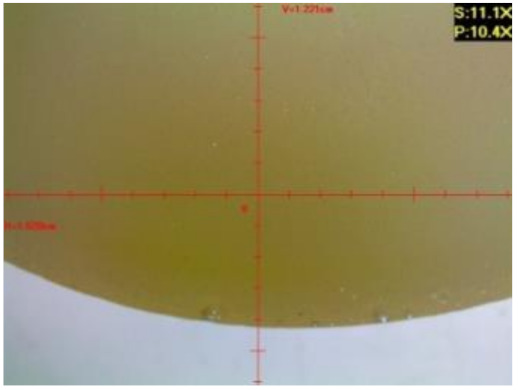
**Environment–aqueous solution of acetic acid 6%**		
RKO6%/1w	RKO6%/1m	RKO6%/3m
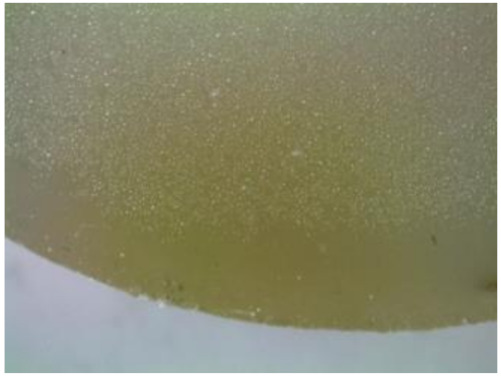	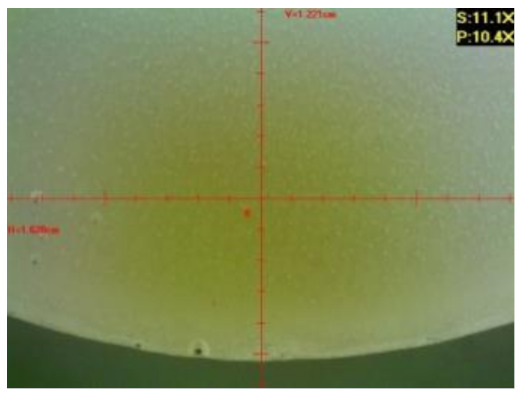	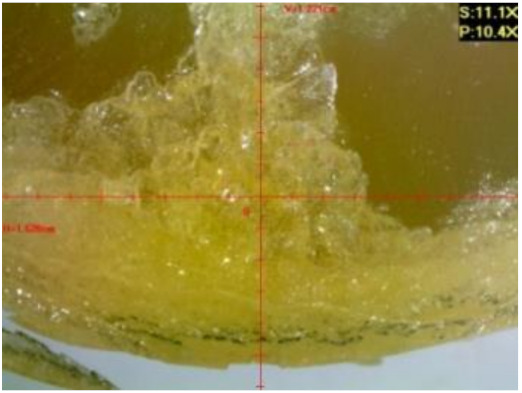
**Environment–aqueous solution of acetic acid 9%**		
RKO9%/1w	RKO9%/1m	RKO9%/3m